# APJ as Promising Therapeutic Target of Peptide Analogues in Myocardial Infarction- and Hypertension-Induced Heart Failure

**DOI:** 10.3390/pharmaceutics15051408

**Published:** 2023-05-04

**Authors:** Daniela Rossin, Roberto Vanni, Marco Lo Iacono, Caterina Cristallini, Claudia Giachino, Raffaella Rastaldo

**Affiliations:** 1 Department of Clinical and Biological Sciences, University of Turin, 10043 Orbassano, Italy; d.rossin@unito.it (D.R.); marco.loiacono@unito.it (M.L.I.); claudia.giachino@unito.it (C.G.); raffaella.rastaldo@unito.it (R.R.); 2 Institute for Chemical and Physical Processes, IPCF ss Pisa, CNR, 56126 Pisa, Italy; caterina.cristallini@cnr.it

**Keywords:** apelin, ELABELA/Toddler/Apela, APLNR/APJ receptor, peptide analogue, cardiovascular disease, myocardial infarction, heart failure, hypertension, angiogenesis, fibrosis

## Abstract

The widely expressed G protein-coupled apelin receptor (APJ) is activated by two bioactive endogenous peptides, apelin and ELABELA (ELA). The apelin/ELA-APJ-related pathway has been found involved in the regulation of many physiological and pathological cardiovascular processes. Increasing studies are deepening the role of the APJ pathway in limiting hypertension and myocardial ischaemia, thus reducing cardiac fibrosis and adverse tissue remodelling, outlining APJ regulation as a potential therapeutic target for heart failure prevention. However, the low plasma half-life of native apelin and ELABELA isoforms lowered their potential for pharmacological applications. In recent years, many research groups focused their attention on studying how APJ ligand modifications could affect receptor structure and dynamics as well as its downstream signalling. This review summarises the novel insights regarding the role of APJ-related pathways in myocardial infarction and hypertension. Furthermore, recent progress in designing synthetic compounds or analogues of APJ ligands able to fully activate the apelinergic pathway is reported. Determining how to exogenously regulate the APJ activation could help to outline a promising therapy for cardiac diseases.

## 1. Introduction

Heart failure (HF) is one of the most common global causes of morbidity and mortality, and its prevalence may rise due to the growing aging population and a variety of risk factors [1]. HF is a chronic clinical syndrome characterized by structural and/or functional cardiac abnormalities, leading to left ventricle (LV) dysfunction. Both hypertension and myocardial infarction (MI) trigger negative cardiac tissue remodelling that, together with the very limited regenerative capacity of cardiac tissue, leads to progressive heart impairment that ultimately compromises cardiac function. The existing HF medications involve surgical and non-surgical techniques used to counteract cardiac deterioration and remodelling, including drug treatments aimed to increase cardiac functionality, decrease hypertension, and/or reduce myocardium extracellular matrix deposition. Although these treatments have decreased the rate of morbidity and mortality, they are, however, uncapable of avoiding ventricular remodelling and progressive fibrosis [2].

The apelin/ELABELA-APJ axis can represent a potential HF therapeutic target and has been shown to play a role in the pathophysiology of both MI and hypertension. However, one of the challenges in using the native apelin and ELABELA (ELA) peptides as therapeutic agents is their short half-life in the blood circulation or extracellular environment. To overcome this limitation, researchers are developing analogues of apelin or ELA that improve their stability and bioavailability. In this review, we explore the recent insights regarding the potential of apelin/ELA-APJ signal activation as therapeutic strategy in hypertension, MI, and heart failure. This review will also highlight the challenges and limitations associated with the development of apelin and ELA peptide analogues, providing an overview of the future direction in this field.

## 2. APJ and Its Endogenous Agonists

### 2.1. APJ

Apelinergic system history began in 1993 with the first description of the apelin receptor (APJ, also known as APLNR) based on its 31% homology with angiotensin II type 1 receptor (AT1R) [3]. Since the homologous sequences are primarily present in the seven hydrophobic transmembrane domains, APJ does not bind to angiotensin II (AngII) and for this reason was considered an orphan receptor for many years. At the same time, tissue distribution of APJ and AT1R is quite similar. APJ is widely expressed in the body and highly expressed mainly in the cardiovascular (CV) system where this receptor is present in cardiomyocytes (CMs), endothelial cells (ECs), and vascular smooth muscle cells (SMCs) [4,5].

The amino acid sequence of this receptor is made of 377 amino acid residues and is well conserved; in fact, the human APJ gene (APLNR) located on chromosome 11q12 displayed more than 90% sequence homology to murine APJ [6]. N-terminal glycosylation of APJ is essential for the proper receptor’s stability, protein folding, and binding to the ligand, while C-terminal palmitoylation favours the association to the cell membrane. Moreover, palmitoylation combined with phosphorylation allows APJ internalisation, dimerization, and the interaction with ligands [7]. APJ is a G-protein-coupled receptor (GPCR), and G proteins are formed by three different subunits (α, β, and γ) and are classified according to the α subunit in four families (i.e., Gαi/o, Gαq/11, Gαs, and Gα12/13) that trigger several different signalling pathways [8]. Upon C-terminal phosphorylation of APJ by GPCR kinases, β-arrestin is recruited and inhibits APJ activation by promoting its internalization [9]. It is also interesting to note that in the heart APJ may act as a mechanosensor for stretch through recruitment of β-arrestin [10].

The role of APJ resulted as pivotal in the cardiovascular system. During embryogenesis, APJ is expressed in mesoderm and APJ deficiency-caused defects in coronary vessel development [11,12]. In adults, APJ activation led to vasodilatation resulting in lowered blood pressure, angiogenesis, haemostasis, and anti-thrombotic effects. In the heart, it increases conduction velocity within CMs, has antiarrhythmic properties, and reduces myocardial hypertrophy and fibrosis [8].

APJ expression increases after ischaemia through the hypoxia-inducible factor-1 (HIF-1a) pathway [13].

### 2.2. Apelin

**Figure 1:**
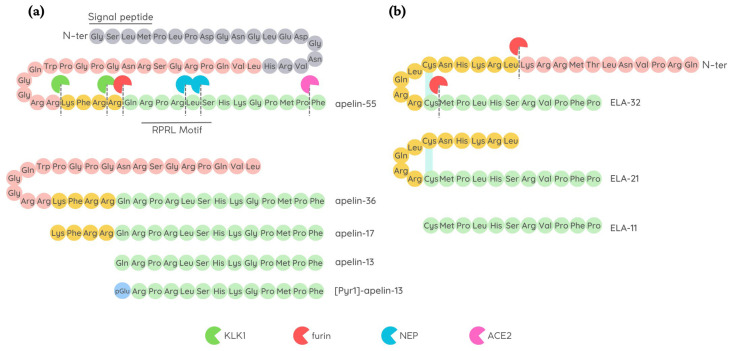
Amino acid sequence of apelin and ELABELA (ELA) peptides. (**a**) Schematic representation of apelin-13, apelin-17, apelin-36, and [Pyr1]-apelin-13 that may all be produced by further processing of apelin-55; all the isoforms have the ability to bind APJ. Angiotensin-converting enzyme type II (ACE2), furin (also known as PCSK3), kallikrein (KLK1), and neprilysin (NEP) degrade native apelin isoforms, thus affecting their bioavailability; (**b**) Schematic representation of ELA peptide maturation by furin convertase to generate ELA-21 and ELA-11 fragments. Disulfide bridges are shown as light blue lines.

Apelin was the first discovered natural endogenous ligand of APJ. Apelin was initially isolated from a cow’s stomach in 1998 by Tatemoto and co-workers [14], and afterwards, the presence of apelin mRNA has been detected in numerous tissues and organs, including adipose tissue, brain, liver, kidneys, skeletal muscles, heart, vessels, and lungs [15,16,17]. Apelin is encoded by a gene located on chromosome Xq 25–26 in various animal species including humans, mice, rats, and cows [16]. Apelin is initially produced as a preproprotein containing 77 amino acids, with the active sequence located in the C-terminal region. The removal of 22-unit signal peptide from N-terminal preproprotein generates the proprotein, also known as apelin-55 [18]. Endopeptidases further cleave apelin-55 into bioactive isoforms called apelin-36, -17, or -13, accordingly to the number of amino acid residues (Figure 1). Among them, the shorter peptides apelin-17, -13, and the pyroglutamate modified form of apelin-13 called [Pyr1]-apelin-13 have a higher affinity for APJ [14], while fragments shorter than 10 amino acids are biologically inactive [19]. Difference in isoform distribution was also observed; for example, [Pyr1]-apelin-13 is predominant isoform in the heart and plasma due to its resistance to degradation by peptidases [20,21], while apelin-36 is mainly detected in the lung, testis, and utero [22].

Apelin isoforms display a brief half-life in vivo, lasting less than 5 min [23,24] due to their rapid breakdown by different proteases. Angiotensin converting enzyme II (ACE2) is a carboxypeptidase that was firstly identified as apelin-degrading enzyme. ACE2 cleaves the Pro12–Phe13 peptide bond at C-terminal level, generating inactive apelin isoforms [22,25,26]. Later, other two cleavage sites within the apelin sequence were identified in apelin-13: Arg4–Leu5 and Leu5–Ser6 at C-terminus (RPRL motif), which are cleavage sites of a zinc-dependent metalloprotease called neprilysin (NEP) [27], thus abolishing apelin binding to APJ [28]. Recently, prolyl carboxypeptidase (PRCP), another protease capable to hydrolyse [Pyr1]-apelin-13, has been identified in endothelial cell membrane [29] while the action of plasma serine protease kallikrein B1 (KLKB1) on the first three N-terminal residues of apelin-17 result in the production of an inactive C-terminal 14-mer [30].

Apelin/APJ axis plays a pivotal role in maintaining CV homeostasis [8,31].

In healthy humans, plasma apelin account for 0.26 ± 0.03 nmol/L concentration, and this low level would suggest a paracrine and autocrine activity [32]. However, hypoxia conditions may raise the plasma level of apelin, and this synthesis is mediated by HIF-1 binding to the apelin gene [33,34,35].

### 2.3. ELABELA

The discrepancy found in the foetal phenotypes between apelin knock-out and APJ-deficient mice, with observation of improper cardiac vessel formation and prenatal mortality, respectively, later led to the identification of Apela/ELABELA/Toddler (hereafter referred to as ELA) as a second APJ ligand [36,37].

It is encoded by the Apela/ELABELA/Toddler gene on chromosome 4q32.3, which is activated since the early embryonic stage. Apela gene encodes 54 amino acids pre-proprotein from which the active peptides ELA-32, -22, -21, and -11 (referring to the number of remaining amino acid residues at the C-terminus) are generated [36,38] (Figure 1). The C-terminal 13 residues of ELA are highly conserved across species and are required for APJ binding and the consequent activation of Gi protein alpha subunit (Gαi) as well as β-arrestin signalling pathways [38,39].

ELA-32 and ELA-11 differ in their membrane-interactive properties, providing a potential mechanism for distinctive signalling outcomes [40].

ELA is highly expressed in embryos, but is also present in some adult tissues, i.e., heart and blood vessels [32,41,42]. Like apelin, ELA displays a short half-life in plasma, and furins have been proposed as possible proteases able to cleave ELA-55 in two conserved di-arginine motifs [37,38].

The absence of the ELA gene causes early abnormalities in heart development, as observed in APJ-knockout animals [37], thus highlighting a crucial role of ELA in cardiac development and also leading to suggest a potential role of ELA in cardiac regeneration. In the rodent heart, ELA is predominantly expressed in the non-CM fraction, suggesting that ELA expression mainly occurs in fibroblasts and endothelial cells [42]. Paracrine and autocrine activities were suggested because of its low plasma level in humans (0.34 ± 0.03 nmol/L) [32], while in mouse heart, samples obtained 4 weeks after MI ELA expression resulted enhanced in the left ventricle of about 6 folds compared to control [42].

Due to the rather recent discovery of ELA, there is still much to find out about its endogenous isoform functions and their interaction with APJ.

### 2.4. Physiological Ligand Activities in CV System

The apelin/ELA-APJ pathway is already involved in the embryonic heart development, even though the two endogenous ligands take part to this process at different time points or areas of the foetal heart as already reviewed by Kuba and co-authors [43]. In the adult CV system, upon binding to the APJ receptor, apelin and ELA exert mostly similar biological effects.

After APJ binding, both apelin and ELA, at nanomolar concentration, exert positive inotropic effect on the in vivo rat hearts with comparable enhancement in the maximal rate of rise of left ventricular pressure (dP/dtmax), fractional shortening (c), heart rate, and cardiac output. Moreover, they reduce both left ventricular end diastolic pressure (LVEDP) and left ventricular end systolic pressure (LVESP), the latter likely due to a reduction of afterload (i.e., the resistance to cardiac output) caused by vasodilation [32,38,44]. Apelin expression appeared to be crucial for proper heart function as proved by the studies of Kuba and colleagues where the lack of apelin gene caused systolic dysfunction and the gradual decline in cardiac contractility, despite the absence of histological abnormalities [45]. Apelin-induced increase in contractility was extensively studied and showed the involvement of several pathways, among which the activation of pro-survival kinases PKC and ERK1/2 that favour the opening of sodium (NHE-1) and Ca^2+^ (NCX) channels [46,47]. Regarding ELA-dependent inotropic effects, ELA-32 was studied by Perjés and colleagues who attributed the increase in contractility to MEK1/2-ERK1/2 but not to PKC, whereas the apelin inotropic effect was mediated by both ERK1/2 and PKC [42]. However, the ELA-mediated inotropic effect was not completely suppressed after the inhibition of the MEK1/2–ERK1/2 pathway [42], thus suggesting that other additional pathways can be activated by this peptide. Further investigation will be needed to clarify this issue. Remarkably, in the healthy hearts, apelin treatment exerted a moderate inotropic effect that only lasted few minutes, while the inotropic effect resulted as particularly marked when administered in failing hearts [48,49].

More recently, a significant increase in lusitropic effect in response to apelin treatment was observed in mouse apelin knock-out CMs [50]. Apelin-induced positive lusitropic effect can be attributed to the preservation of SERCA activity avoiding the enhancement of intracellular calcium level, which may cause calcium overload-induced arrhythmogenesis [50,51].

Apelin and ELA are also vasoactive peptides with different mechanisms of action. Their administration results in a reduction in vascular resistance with consequent increase in blood flow accompanied by arterial pressure decrease. Apelin usually induces endothelial-induced vasodilation mediated by NO release via Akt activation [23,52,53], while in the case of endothelial disfunction, the peptide acts directly on smooth muscle cells triggering the opposite effect [54,55]. On the contrary, ELA-induced vasorelaxation did not require NO release [37]. Recently, the mechanisms of ELA-induced vasodilation have been elucidated by Sahinturk and collaborators who reported that endothelial-dependent vasorelaxation is mediated by prostanoids, while AMPK, PKC, and calcium-activated potassium channel activation was observed in the endothelial-independent pathway [56]. It is likely that beside the apelin/ELA-mediated increase in contractility, these peptides improved cardiac function by inducing vasodilation. In fact, the lowered afterload and the increased venous return (preload) allowed the heart to enhance the stroke volume. These APJ ligand effects can be useful in hearts with progressive function deterioration.

Notably, both peptides are involved in the angiogenic process (see below). Moreover, their ability to attract and promote migration of endothelial cells led to consider apelin and ELA as chemotactic peptides in the angiogenic process [57,58].

APJ knockout mice exhibit abnormal body fluid balance [59] demonstrating the involvement of apelin and ELA in water homeostasis. Indeed, they reduce water intake, and apelin also increases water excretion [60,61]. Moreover, apelin also resulted as involved in energy metabolism by increasing glucose uptake by tissue cells and insulin sensitivity and mitochondrial bioactivity, as recently reviewed by Hu and collaborators [60].

## 3. Cardioprotective Role of APJ Endogenous Ligands

In recent years, a growing body of evidence has highlighted the potential of apelin and ELA as therapeutic targets for the treatment of CV diseases, such as hypertension, ischaemic heart disease, and heart failure.

### 3.1. Protection against Hypertension

Hypertension is a chronic medical condition in which the long-term high arterial blood pressure represents a relevant variable risk factor for cardiovascular disease morbidity and mortality because of increased incidence of MI and heart failure development [62,63]. Indeed, the persistence of high blood pressure induces LV hypertrophy and remodelling as adaptive response to cardiac output resistance [64].

The renin–angiotensin system (RAS) is a key pathway in the development and progression of hypertension. AngII, upon binding to AT1R, exerts a vasoconstrictor effect that plays a physiological role in arterial blood pressure control through the RAS. However, AngII–AT1R axis hyperactivation commonly leads to development and progression of hypertension [65]. AngII is formed starting from liver-produced angiotensinogen precursor which is cleaved by renin into Angiotensin I which in turn is converted to AngII through the catalytic activity of the angiotensin converting enzyme (ACE). Conversely, ACE2 negatively regulate RAS through the conversion of AngII into Ang(1–7), resulting in blood pressure reduction [66].

**Figure 2:**
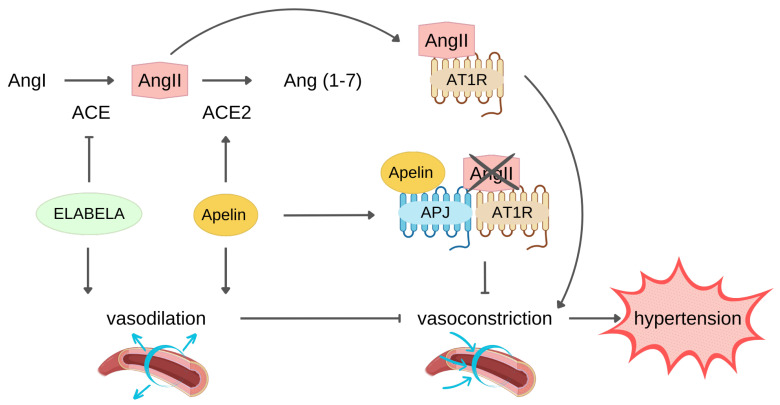
ELABELA and apelin protect against hypertension counteracting the renin-angiotensin II system (RAS) with different mechanisms. Angiotensin Converting Enzyme (ACE) converts Angiotensin I (AngI) to Angiotensin II (AngII), which binds Angiotensin II Receptor Type 1 (AT1R) and induces vasoconstriction, leading to hypertension. Endogenous ELABELA and apelin neutralize AngII-induced vasoconstriction through their direct vasodilator effect. Both ligands exert vasodilation through AngII level reduction: ELABELA downregulates ACE expression, limiting AngII production, while apelin boosts the expression of ACE2 which hydrolyzes AngII to Ang(1–7). The binding of apelin to its receptor APJ leads to the heterodimerization of APJ and AT1R, decreasing the AT1R availability for binding AngII, thus inhibiting the AngII-AT1R signalling pathway that leads to hypertension.

The apelin/ELA-APJ system can affect hypertension counteracting RAS (Figure 2).

Indeed, apelin/ELA and AngII exerted an opposite effect on vessels. In this regard, it has been reported that administration of either apelin or ELA had a blood pressure-lowering effect, potentially indicating a therapeutic role of these peptides in reverting hypertension, thus reducing the risk of developing HF [67,68]. In addition, apelin is capable of boosting ACE2 expression, and this is another mechanism by which it counteracts RAS. In fact, Sato and colleagues pointed out that apelin improved ACE2 promoter activity in neonatal CMs in a dose-dependent manner [69]. In line with the in vitro results, apelin KO mice also showed downregulation of ACE2 expression and, consequently, low ACE2 protein level [69,70]. Of note, ACE2, which primarily acts on AngiII, has a role in apelin metabolism because it can cleave apelin into inactive isoforms, though it is unable to degrade ELA [23,66,67]. Conversely, ELA did not affect ACE2 expression, but it reduced angiotensin-induced blood pressure via downregulation of ACE expression.

Another apelin mechanism opposing RAS consists of heterodimer formation by APJ and AT1R interaction, resulting in inhibition of AngII binding to its receptor and signalling pathway activation [71].

Protracted hypertension induces remodelling of the heart which consists of cardiac hypertrophy and deposition of collagen fibres between the CMs, namely interstitial fibrosis. This morphological alteration also contributes to cardiac dysfunction, and indeed, CMs develop hypertrophy as adaptation to elevated cardiac workload, with the aim of maintaining left ventricle ejection fraction (LVEF) unchanged, at least at the beginning of the pathological condition. This protracted cardiac remodelling can give rise to arrhythmogenesis and HF over-time [72].

The link between the apelinergic pathway and AngII RAS pathways was studied in apelin−/−mice through subcutaneous infusion with AngII for two weeks. AngII treatment caused increased cardiac dysfunction, hypertrophy, and fibrosis and enhanced ACE/ACE2 as well as TGF-β expression in apelin−/−compared to WT mice [73]. It was also observed that the loss of apelin facilitated AngII-induced injury pathways, leading to enhanced pathological hypertrophy and myocardial fibrosis [70,74]. Furthermore, apelin-deficient mice showed downregulation of ACE2, increased superoxide production, and augmented NADPH oxidase activity [70].

In another animal model, AngII-induced hypertension yielded an increase in myocardial fibrosis and inflammation. Moreover, AngII lowered apelin and ELA levels in both rat and primary neonatal rat cardiac fibroblasts, and these results were in line with the low level of these peptides in hypertension patients [75]. The adverse remodelling was shown to be mediated by miR-122p, as the treatment of primary neonatal rat cardiac fibroblasts with both AngII and the inhibitor of miR-122p abolished the AngII-induced downregulation of APJ ligands [75]. Interestingly, pre-treatment of cardiac fibroblasts with apelin or ELA prevented the enhancement of inflammation and fibrosis markers. These authors also highlighted a link between APJ ligand level and cardiac fibrosis [75].

On the other hand, the animal treatment with apelin-13 could counteract AngII-induced fibrosis via TGF-β1/SMAd2/α-SMA and preserved the homogeneity of electrical conduction [76].

In in vivo studies, the AngII-dependent ELA reduction increased cardiac hypertrophy, fibrosis, and dysfunction in murine heart, while ELA administration reduced cardiac fibrosis, ultrastructural injury, lipid peroxidation, and inflammation, thus improving cardiac function [77]. Moreover, ELA mice administration for two weeks after transaortic constriction (TAC) promoted ACE expression downregulation and reduced cardiac fibrosis, promoted the maintenance of FS rate, and suppressed the expression of cardiac hypertrophy markers, such as BNP, ANF, and β-Myhc [78].

Notably, ELA and apelin circulating levels were found decreased in patients with hypertension [79,80,81]. Additionally, Baysal and colleagues discovered that hypertensive individuals with impairment in left ventricular systolic and diastolic performance displayed low plasma apelin level that increased following one month of antihypertensive medication [82]. Further, concerning the ELA level, it was observed that reduced ELA plasma concentration was associated with atrial fibrillation in hypertensive patients [83]. Among the HF patients, those with higher level showed a more preserved LVEF and better outcomes [84]. Furthermore, low apelin and ELA expression was found in patients with pulmonary arterial hypertension [32].

**Figure 3:**
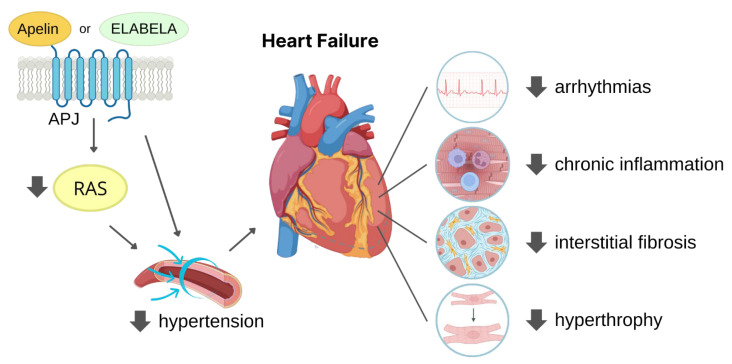
Dual mechanism of ligand-activated APJ in reducing hypertension-induced heart failure. The figure illustrates the two ways in which APJ activation can reduce cardiac overload-induced heart failure. Firstly, by reducing renin-angiotensin system (RAS) activation, APJ lowers blood pressure, thus decreasing cardiac afterload. Secondly, activated APJ directly acts on the heart, decreasing the risk of developing arrhythmias chronic inflammation, interstitial fibrosis, and cardiac hypertrophy, leading to improved cardiac function.

Taken together, these findings suggest that APJ ligands have significant potential as therapeutic strategy to counteract hypertension and the related complications (Figure 3).

### 3.2. Protection against MI

MI is a critical cardiovascular event associated with high morbidity and mortality in which blood flow blockade prevents the O_2_ supply to the cardiac tissue. The restoration of blood flow, via thrombolytic therapy, primary percutaneous coronary intervention, and coronary artery bypass grafting (CABG), is essential for cardiac tissue salvage. However, after reperfusion, an exacerbation of myocardial damage takes place. For this reason, it is more correct talking of ischaemia-reperfusion (I/R) injury that consists of a series of events including cell death, inflammation, oxidative stress, calcium overload, and paradoxical pH that lead to cardiac tissue damage as well as impaired heart function (i.e., stunning of myocardium) [85]. Notably, it has been estimated that about 30% of patients develop I/R injury which seriously affected patient prognosis [85,86].

#### 3.2.1. I/R

**Figure 4:**
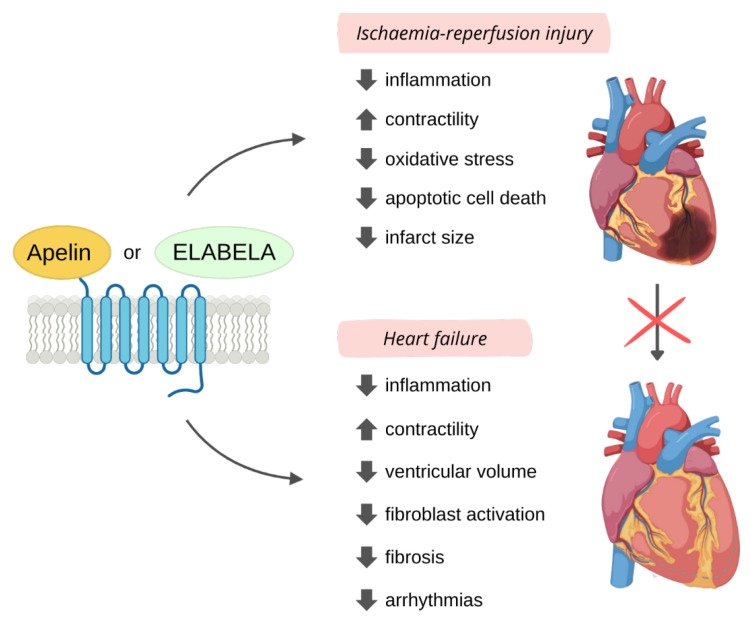
Multiple cardioprotective effects of ligand-activated APJ in MI-induced heart failure. Upon ligand binding, activated APJ counteracts ischaemia-reperfusion injury, including reduction of infarct size, inflammation, oxidative stress, and apoptotic cell death, as well as enhancement of cardiac contractility. APJ ligands also contrast heart failure developed after MI by increasing cardiac contractility and reducing ventricular volume, inflammation, fibroblasts activation, fibrosis, and arrhythmia.

In recent years, apelin and ELA have emerged as potential therapeutic tools for myocardial I/R injury limitation, as they have been shown to exert protective effects against various pathological processes associated to this condition (Figure 4).

Indeed, Wang and co-workers reported that both ex vivo and in vivo hearts underwent I/R are more susceptible to infarction and compromised cardiac function in the absence of apelin [87].

As for the involved pathways, several articles reported that APJ ligands exert cardioprotection against I/R mainly triggering PI3K-Akt-NO and MEK 1/2-ERK1/2 signalling pathways [85,88,89,90,91,92,93].

An additional study performed in ex vivo rat hearts which underwent 30 min of ischaemia and 2 h of reperfusion reported that apelin-13 cardioprotection also involves PTEN inhibition via Src kinase-mediated phosphorylation, thus abolishing PTEN activity which hinders PI3K/Akt pathway activation [94]. Accordingly, in diabetic mice, the key role of PTEN inhibition resulted as essential to preserve the cardioprotective effect of post-conditioning, a mechanical manoeuvre consisting of several brief ischaemia and reperfusion cycles performed at the onset of reperfusion, to which the diabetic animals are not responsive due to a significantly higher expression of PTEN after I/R [95].

The cardioprotective effects induced by APJ ligands against I/R damage mainly consist of limitation of infarct size and myocardial contracture along with improvement of post-ischaemic contractile recovery, as highlighted in both in vivo and ex vivo models. More specifically, the ischaemia causes necrosis of myocardial tissue, and the extension of infarct size is mostly correlated to the duration of ischaemia [96]. As said, the prompt reperfusion is essential to limit myocardial necrosis, but paradoxically, it exacerbates the tissue damage. The activation of APJ receptors via administration of apelin and ELA was demonstrated to reduce the infarct size. In fact, apelin-13 administration during the first 20 min of reperfusion reduced the extension of infarct size from 60% to 30% in ex vivo isolated hearts [94], whereas it was ineffective when administered 20 min before I/R [88]. Conversely, other recent investigations showed that apelin succeeded in decreasing infarction extension when given before ischaemia. In fact, apelin-13 infusion for 5 days before I/R induction by LAD occlusion (30 min) in ex vivo rat hearts lowered infarct size from 45% to 30% of the risk zone [71] and apelin-13 administration for 4 weeks before I/R limited the infarct size in a mouse I/R model (17% vs. 38% in control hearts) [97]. While the administration at the beginning of reperfusion (post-ischaemic) might be used in the clinical setting, such as during a percutaneous intervention or coronary artery bypass grafting, pre-ischaemic treatment to protect the myocardium against I/R injury, however, is unfeasible in the clinical practice due to ischaemic event unpredictability.

Moreover, ELA reduced the infarcted area of about 50% compared to control hearts, when injected into peritoneum for 4 days after I/R [98].

APJ ligand-induced protection against infarct size extension was also confirmed by studying biomarkers usually employed for MI diagnosing, such as creatinine kinase myocardial band (CK-MB) isoform, cardiac Troponin levels, and LDH release in plasma serum, which resulted as decreased by both native peptide administration [71,94,97]. It is well-known that mitochondrial permeability transition pore (mPTP) plays a pivotal role in cardiac damage caused by I/R, because its opening causes dissipation of mitochondrial membrane potential, elevation of reactive oxygen species (ROS) production, calcium overload into the cytoplasm that contributes to osmotic swelling, and release of proapoptotic protein (i.e., cytochrome-c) which gives rise to cell death. For this reason, the inhibition of mPTP opening is crucial for limiting cell death [99]. In a model of I/R rat heart, Yang and collaborators proved the capability of apelin-13 in hindering mPTP opening through the activation of PI3K-Akt-GSK3β signalling pathway [89]. Other in vitro studies pointed out that apelin-13 suppressed the alteration of mitochondrial membrane potential, mitochondrial ROS generation, and apoptosis in both H9c2 cardiomyoblasts and neonatal CMs exposed to hypoxia-reoxygenation protocols [89,97], resulting in a reduction of apoptotic cell death rate. Likewise, ELA also displayed an improvement in mitochondrial dysfunction in both in vitro and in vivo models of I/R injury. In fact, the systemic administration of ELA at the beginning of reperfusion, which followed 30 min of LAD occlusion, prevented mitochondrial morphology alteration and attenuated ROS release [85]. ELA ameliorated apoptosis and oxidative stress triggering the PI3K-Akt-NOS signalling pathway, as evidenced by the reduction in cytochrome-c and caspase-3 release along with the upregulation of anti-apoptotic Bcl-2 and downregulation of pro-apoptotic Bax in rat and mouse hearts that underwent I/R [85,97,100,101].

Furthermore, evidence of anti-inflammatory action exerted by apelinergic system in cardiac tissue was pointed out in apelin-deficient hearts where both monocyte infiltration along with abundant cytokines and interferon release were observed [102]. Even ELA pre-treatment had anti-inflammatory effect via modulation of IL-10, IL-1β, IL6, and CXCL1 [101]. Unlike apelin, the possible inhibition of GSK3β by ELA is still unknow in the heart. However, in mesenchymal stem cells (MSCs) exposed to hypoxia, ELA stimulated the activation of PI3K-Akt and ERK signalling pathway, avoiding mitochondrial disfunction [103]. Accordingly, apelin-13-treated MSCs injected after MI in the border zone showed lowered mitochondrial dysfunction and apoptosis attributed to the activation of ERK signalling pathway [104].

Concerning contractile dysfunction, the part of myocardium that is not irreversibly injured suffers from a reversible mechanical dysfunction, namely hypocontractility or stunning, which also persists after the restoration of blood flow after an ischaemic episode. Calcium overload lowers responsiveness of myofilaments to calcium, and sarcoplasmic reticulum dysfunction-induced excitation–contraction uncoupling is considered the mechanism accountable for this ventricular contractile dysfunction [105]. Several articles confirmed the efficacy of apelin-13 treatment before ischaemia (4 weeks before MI) in improving contractility in post-infarcted hearts, as evidenced by echographic assessment of the LVEF and left ventricle fractional shortening (LVFS) recovery after 6 h of reperfusion [97]. This improvement in cardiac function was also confirmed in an ex vivo rat model, where the intraperitoneal administration of apelin-13 (5 days) before transient occlusion of LAD determined LVDevP and dP/dt max recovery, accompanied by an improvement in cardiac relaxation as revealed by the reduction of LVEDP that is an index of contracture, in agreement with previous results from Folino et al. [71,94]. It should not be overlooked that the mechanical recovery might also be supported by higher number of viable CMs, due to infarct size limitation, as previously discussed.

During and immediately after MI, ventricular arrhythmia can occur, leading to sudden cardiac death. More than 10% of all acute MI cases have serious ventricular arrhythmias before admission to hospital, which lowers the survival chances for these patients [106]. In this regard, the injection of apelin before MI enhanced PI3K-dependent potassium channel (IK1/Kir2.1) currents and reverted the ischaemia- and hypoxia-induced resting membrane potential depolarization and prolongation of QT interval on the ECG, which is thought to be the pro-arrhythmogenic mechanism following MI [107]. These results are in line with a previous study in which electrophysiological features of cardiac tissue were affected by apelin level [108]. In addition, the peptide level also influenced the anti-arrhythmic effect of pharmacological therapy in patients [109].

Only a few studies investigated the cardioprotective role of ELA [85,92,97,100], though its effectiveness against I/R injury was confirmed in other organs, such as kidney, brain, and lung [110,111,112,113].

#### 3.2.2. Adverse Remodelling

The phenomenon of adverse cardiac remodelling following MI involves an intricate interplay between various cellular and extracellular components of the heart tissue, thus resulting in alterations of the cardiac architecture, size, and shape that compromise heart function, increase the risk of developing HF, and generally associate with long-term poor clinical outcome [114,115].

The association between cardiac fibrosis and poor prognosis may be attributed to extracellular matrix (ECM) deposition along with exacerbated long-lasting inflammatory response that negatively affects systolic and diastolic function and promotes arrhythmic events [116,117,118,119].

However, a certain degree of myofibroblast activity is essential for cardiac repair after MI because it preserves myocardial structural integrity, as proved in an experimental model with myofibroblast removal in which animal death was due to an inadequate scar formation and consequent ventricular wall rupture [120], yet the protraction of remodelling drives to HF.

In cardiac remodelling, starting a few hours from acute coronary occlusion, necrosis of CMs together with inflammation are the primary processes. During this phase, inflammatory cells and fibroblasts are recruited into the ischaemic area. Subsequently, cardiac fibroblasts and other cells, such as epicardial epithelial cells or endothelial cells, can undergo transdifferentiation into myofibroblasts, which are not present in the healthy myocardium. Myofibroblasts display characteristics of both fibroblasts and smooth muscle cells and are capable to synthesize large amounts of ECM proteins [121,122,123]. Myofibroblasts mainly take part in ECM degradation by releasing remodelling enzymes (such as MMPs), and, at the same time, they increase new fibrotic collagen deposition. These events lead to the replacement of dead CMs with fibrotic scar with tensile strength, thus providing a mechanical support to the injured tissue that prevents ventricular wall rupture. At the beginning, these processes are adaptive, whereas their protraction over time leads to an exaggerated ECM deposition with consequent alteration of cardiac morphology and heart size. Crosslinking formation in the extracellular matrix accounts for scar maturation accompanied by partial myofibroblast depletion by apoptosis, while the resistant ones perpetuate the same process [124,125].

A dramatic change in protein levels of both APJ and its ligands was observed post-MI. Tatin and collaborators reported a significant increase in APJ gene expression in cardiac tissue only in the late phase, one month post-MI [126,127,128]. Of note, the same authors reported a different trend of apelin gene expression compared to APJ in the infarcted area of mouse hearts. Indeed, this ligand was characterized by an earlier increase, two days post-MI, followed by a reduction to basal levels seven days and one month after MI [126]. The increase in apelin levels in the early period after MI has also been recently confirmed in a clinical study, reporting a significant six-fold increase in apelin in MI patients at hospital admission compared to the control group [35]. These data are in line with Krasniqi and collaborators who reported that high apelin levels within seven days post-MI were correlated to low rates of developing major adverse cardiac events (MACE) in a clinical study [129]. It should be kept in mind, however, that the spontaneous increase in apelin levels in the first days after MI was not sufficient to fully protect the hearts. For this reason, an exogenous administration of APJ ligands might be required to efficiently prevent heart damage and restore cardiac function.

Unlike apelin, the expression of ELA and APJ increased in murine LV one month post-MI, and these findings were associated with a major preservation of LV systolic function [42], although earlier time points were not investigated by these authors. However, a clinical study reported a 6-fold increase in ELA circulating level in patients 90 minutes after MI compared to healthy subjects [130], thus suggesting the possibility for an early post-MI increase also for ELA. Notably, no other evidence regarding the ELA levels in post-MI hearts has been published, and further efforts should be made to better investigate the course of this APJ ligand level after MI.

In any case, post-MI changes in apelin and ELA expression over time and their beneficial effects along with improved outcomes observed in patients showing higher ligand expression suggested their important role in cardioprotection post-MI (Figure 4), thus leading to overexpression or exogenous administration studies.

The overexpression of apelin in the myocardium had a protective effect by preventing cardiac fibroblast activation via sphingosine kinase 1 (sphk1) inhibition, reducing the levels of TNFα and IL1β proinflammatory mediators, and decreasing CD68+ macrophage infiltration in the ischaemic heart [126]. Afterwards, the ability of apelin to counteract inflammation was recently confirmed in MI rats, where apelin-13 administration for 4 weeks also prevented cardiac fibrosis by inhibiting the increase in collagen I, collagen III, and TGF-β levels [128]. Zhang and co-workers found that intraperitoneal injection of apelin-13 for 4 weeks after LAD ligation-induced MI, besides reducing TGF-β and NF-kB pro-inflammatory mediators, reverted EMC-degrading enzymes MMP-2 and -9 in rat hearts. It resulted in a reduction in histopathological damage and cardiac fibrosis percentage as compared to the MI rat group [131]. Moreover, the long-term apelin overexpression reduced the expression of hormones associated to HF, i.e., atrial natriuretic peptide (ANP) and brain natriuretic peptide (BNP), in rat hearts 6 weeks after MI. Since apelin treatment is capable of reverting the alteration of MI-induced MMP level, it is possible that upon binding to APJ, this peptide might also suppress the progression of maladaptive cardiac remodelling process besides preventing it. Further investigations are needed to clarify the effectiveness of apelin in blocking progression of maladaptive remodelling when administrated in chronic MI experimental models.

Regarding ELA, a reduction in type I and III collagen was shown after ELA treatment of H9c2 cells underwent hypoxia-reoxygenation in order to mimic I/R in vitro [85]. These data were also confirmed in in vivo experiments where ELA treatment significantly decreased collagen deposition two weeks after MI, thus limiting the extension of fibrosis in rat hearts [93]. However, Yu and co-workers showed that activation of PI3K-Akt-NOS signalling pathway was responsible for the lowering of type I and III collagen protein expression, whereas Rakhshan and collaborators reported the involvement of ERK 1/2 phosphorylation only [85,93]. Additionally, Rakhshan et al. revealed a reduction in interstitial fibrosis that might result in an improved heart function, yet unfortunately, cardiac functionality was not investigated by the authors [93]. Altogether, these papers highlighted that both ligands are capable of limiting profibrotic activity, thus counteracting the development of HF.

In post-MI remodelled cardiac tissue, the deposition of collagen fibres between CMs can also alter gap junction-mediated electrical impulse transmission, resulting in disorganisation of normal microconduction pathways that bear to QT interval prolongation, predisposing to fatal arrhythmias [132,133]. After MI, the recovery of a proper gap junction structure is necessary to improve electrical transmission and coordinated contraction of CMs. Connexin-43 (Cx43) is a key protein forming gap junctions, and a recent study has shown that apelin-13 plays an important role in Cx43 modulation. Non-myocytic cells treated with apelin-13 showed a significant increase in Cx43 through the activation of the PI3K/Akt/mTOR signalling pathway [134]. It is likely that the recovery of gap junction expression and distribution could abrogate ventricular arrhythmias.

Moreover, it should be kept in mind that fibrosis increases the stiffness of the ventricle, thus reducing ventricular compliance. Sustained cardiac tissue remodelling ultimately leads to the failure of cardiac homeostasis and function [135], causing progressive LV dilation (i.e., increase in LVEDV and LVESV) and cardiac dysfunction (e.g., reduced LVEF and LVFS) [120]. In this context, the apelinergic system may contribute to cardiac function recovery after MI. Indeed, Wang and collaborators showed that the absence of apelin can compromise functional recovery in both ex vivo and in vivo models [87]. Moreover, post-MI apelin-13 administration for 4 weeks in rats was found to improve heart function by increasing both LVDevP and dP/dt max along with LVEDP reduction [128,136]. More recently, the improvement in cardiac performance in post-MI rat hearts after 4 weeks treatment with apelin-13 was observed, thus confirming the previous study from Zhang et al. [131]. In particular, Zhong and colleagues showed an increase in the percentage of LVEF and LVFS via echographic assessment [128]. The same authors also reported that apelin-13 reversed the MI-induced alteration of ventricular pressure and volume. In particular, they observed an increased contractility (dP/dt max) and the reduction in LV end-systolic and -diastolic diameter (LVESD and LVEDD), along with LV volumes in systole and diastole (LVSV and LVDV) [128]. These results indicated that apelin can improve cardiac function and limit LV dilation which is correlated with mortality and HF development after MI [137]. Conversely, Tatin and colleagues showed that long-term apelin overexpression, induced by lentivirus injection at the border zone after MI, did not significantly improve cardiac function 6 weeks after MI, but still triggered a relevant decrease in cardiac fibrosis [126]. However, Macrae and collaborators highlighted that APJ is essential for the acquirement of contractile capacity in ESC-derived CMs as well as for counteracting tissue stiffness and collagen deposition in 3D engineered cardiac tissue [138]. These authors claimed that the reduction in cardiac contractility observed in apelin-deficient mice were in line with the low apelin circulating level in HF patients with impaired cardiac function [139].

It is also well-known that interstitial fibrosis, usually associated with hypertension, is also present in viable cardiac tissue distal from infarcted region accountable for ventricular stiffness as well as alteration of cardiac morphology and ventricular size, with consequent deterioration over time of cardiac function [140]. It cannot be excluded that apelin-induced vasodilation can also positively affect the cardiac performance via reduction of afterload against which the heart pumps blood. In this regard, apart from apelin positive inotropic effect, the anti-fibrotic effect of apelin can also contribute to recovery of cardiac function in MI hearts. Regarding ELA, no data on its capability to hinder ventricular dysfunction has been published to our knowledge. Thereby, further investigation on its potential effect on ventricular size and mechanical recovery is required.

Taken together, these recent findings suggested that APJ ligands may favour reverse remodelling, thus supporting the potential role of APJ in regulating post-ischaemic HF by protecting against adverse remodelling.

#### 3.2.3. Vessel Formation

Cardiac tissue damage resulting from MI also impairs coronary circulation and leads to small vessel rarefication in the infarcted tissue [141]. The recovery of a proper cardiac tissue vascularization is necessary for oxygen and nutrient supply to tissue. Cardiac ischaemia does not elicit an efficient angiogenic response [142], and for this reason, vascularization should be fostered with adequate treatment.

Over the last few years, the involvement of the apelinergic pathway in the angiogenic process has been consolidated [68,143], yet how precisely it is involved is still a matter of debate.

Both apelin and ELA binding to APJ receptor are involved in heart and blood vessel formation during embryonic development, as recently reviewed by Liu and colleagues [144]. Specifically, the ELA-APJ axis is related to embryonic vasculogenesis and angiogenesis, while Apelin-APJ signalling is involved in diameter control of vessels during foetal angiogenesis [145,146]. Interestingly, mice receiving hearts from apelin-deficient donors exhibited impaired endothelial repair and increased vascular disease. In apelin knockdown endothelial cells, treatment with apelin enhanced the regenerative capacity of the scratch wound [102]. Apelin- and APJ-deficient mice displayed abnormalities in artery and vein alignment and reduced vascular extension, EC proliferation, and smaller vessel diameter [147]. Experiments using mice with a deletion of the ELA gene displayed aberrant growth and organization of embryonic blood vessels.

Regarding apelin, its role in angiogenesis was highlighted in many tissues, including the myocardium. Suppression of apelin gene expression in MI rats reduced the angiogenic process [148], and the apelin/APJ system was demonstrated to induce sprouting of endothelial cells [136]. The fluorescent labelling of apelin revealed that its expression increased in coronary endothelial cells after MI and was kept high during the active sprouting, while its expression reduced when functional vasculature was re-established [149].

Zhang and collaborators ascribed the improvement in cardiac performance to apelin-induced angiogenesis [136]. Furthermore, this study highlighted an increase in cell proliferation and recruitment as well as tube formation with dose-dependent response to apelin treatment. An enhancement in vascular endothelial growth factor receptors-2 (VEGFR2) also occurred after apelin-13 treatment [136].

Patients with good coronary collateral development exhibited higher serum ELA levels than healthy patients with poor coronary collateral development, which can be linked to the role of ELA in angiogenesis and arteriogenesis [150]. In adult mice, ELA treatment after MI increased the CD31+ cells in cardiac tissue around the infarcted region. These results, together with a reduction of apoptotic cells and increase in LVEF, led authors to highlight the potentially relevant role of ELA to promote angiogenesis and improve cardiac function [151].

It is well-known that VEGF plays a key role in the regulation of blood vessel growth and angiogenesis. VEGF expression is low in physiological conditions, but its release can rise after MI due to inflammation and mechanical tension [152,153], thus suggesting the stimulation of angiogenesis via the activation of VEGF/VEGFR2 signalling after both apelin and ELA treatment [93,154]. Similarly, in mouse post-MI heart after gene therapy via both systemic and intramyocardial AAV-ELA plasmid injection, VEGF/VEGFR2 gene expression up-regulation was seen, in particular around the infarct area [155]. These authors observed a significant increase in angiogenic marker levels such as CD105, vWF, CD31, and Ki67 in the gene therapy group with respect to the control group [155] and suggested that ELA might boost angiogenesis by recruiting more endothelial progenitor cells. These results are in line with a study previously conducted in post-MI diabetic mice in which apelin overexpression attracted endothelial progenitor cells contributing to enhanced vascular density in the ischemic region [156].

Among the signalling pathways involved in the angiogenic process, the Notch signalling system plays a role in heart development and angiogenesis by regulating SMC differentiation and proliferation [157,158]. Notably, the Notch-dependent signalling pathway can be involved in the angiogenic processes induced by both apelin-13 and ELA. In this regard, it was reported that Notch downregulation reduced SMC proliferation and angiogenesis induced by apelin-13 in in vitro treatment [159]. In addition, after AAV-ELA plasmid injection in mouse post-MI heart, increased expression of Jagged1 and Notch was observed in the cardiac tissue around the infarct [155].

In the Notch-related angiogenic handle, CXCR4 is a downstream effector of Notch signalling and mediates Notch-dependent and -independent EC migration [160,161]. Intraperitoneal apelin-13 administration increased the concentration of vascular progenitor cells and promoted angiogenesis through the CXCR4 signalling pathway by increasing myocardial capillary density and enhancing arteriole formation in MI mice models [162]. Notably, a cross-talk between APJ signalling and CXCR4 in ECs was reported, and drug stimulation of CXCR4 can rescue the loss of signalling axis in apelin/APJ-deficient mice as well as restore the associated vascular phenotypes [163].

Regarding the remodelling process, in addition to angiogenesis, the development of lymphatic vessels in the vascularization process should be considered. In fact, a protracted alteration of lymphatics leads to oedema and fibrosis [164].

APJ receptor expression was present in cardiac lymphatic vessels growing in the infarcted area 6 weeks post-MI, while this was not detected in normal mouse hearts [126]. These data underline that the APJs are strongly spatially and temporally regulated in the lymphatic endothelium. Apelin signalling regulated lymphatic development [165], and apelin expression was associated with cardiac lymphatic vessels [126]. In post-MI apelin-deficient mice, cardiac lymphatics exhibited a dilated morphology and increased infiltration of CD68+ macrophages, leading to an increase in proinflammatory cytokine expression [126]. Moreover, this study also reported an unexpectedly high expression of lymphangiogenic factors (i.e., VEGF-C and D), which led to remarkable lymphatic vessel density enhancement in infarcted hearts. Conversely, apelin overexpression promoted the maturation of developing lymphatic vasculature after cardiac ischaemia by preventing the alteration in cellular junctions as well as reducing the number and dilation of lymphatic vessels, thus resulting in regular morphology and well-shaped organization [126]. To the best of our knowledge, whether ELA affects the lymphatic system has not been defined yet.

APJ activation is a promising strategy for stimulation and regulation of vascular regeneration in cardiovascular diseases.

## 4. APJ Peptide Agonists

Modified peptides can be designed to specifically bind a target receptor. Peptide modifications might modulate protein–protein interactions to improve target selectivity and affinity that play crucial roles during the persistent interaction between proteins. Furthermore, compared to small molecules and antibodies, modified peptides have the important advantage of causing a minor immune response [166]. The small size of APJ peptide ligands makes them easy to manipulate.

In this chapter we report the recent updates and progress in APJ peptide agonists designed for cardiovascular disease treatment focusing on the regulation of the main effects following the modification of their chemical structure.

### 4.1. APJ Endogenous Ligand Main Chain Modifications

The well-known short in vivo half-life that characterises both apelin and ELA poses a hurdle to their clinical translation. Many studies thus aim to develop more stable APJ ligands, based on either apelin or ELA peptide structure, through many different approaches, such as main-chain residue removal/addition, palmitoylation, and cleavage site modification of the several proteases involved in the degradation of parental peptides to obtain longer half-life in blood circulation as well as in tissues, trying to maintain at the same time apelin- or ELA-like cardiovascular effects (Table 1 and Table 2).

#### 4.1.1. Modification of Apelin Cleavage Sites

The peptidase ACE2 acts as a major negative regulator of apelin action in the vasculature and the heart. ACE2 can remove Phe13 at the C-terminal from apelin, thus reducing apelin capability in lowering blood pressure and protecting against heart damage [25].

Since one of the primary causes of apelin-13 short half-life is the breaking of the link between Pro12 and Phe13 by ACE2, it would seem to be quite beneficial to modify this region of the sequence. It was reported that analogues synthesized carrying palmitic acid at N-terminus and aminoisobutyric acid (Aib) at position Pro12 strongly preserved the peptide half-life for 29 h in rat plasma [171].

C-terminal Phe13 residue of apelin is also crucial in determining its affinity for APJ [167]. The replacement of Pro12 and Phe13 residues in apelin-13 with conformationally constrained amino acids significantly increased both binding affinity and peptide stability in plasma [168]. Moreover, the Pro12-Phe13 substitution with Aia-Phe or 1Nal-α,α-dibenzylglycine strongly activated the Gα12 pathways, resulting in higher affinity, resistance to ACE2 cleavage, and enhanced in vitro and in vivo pharmacokinetics [169].

Another study showed that the biased analogues produced by replacing the C-terminal Phe13 of apelin-13 with either (L-α-Me)Phe or p-benzoyl-L-Phe (Bpa) induced APJ functional selectivity for Gαi proteins. While native apelin-13 significantly increased cardiac contractility in isolated as well as in vivo rat hearts, the two biased apelin analogues unfortunately did not show effect on cardiac contractility in healthy rats, even if they displayed similar binding activity to apelin. However, the authors reported that these analogues can reduce hypertrophy and fibrosis in a sympathetic hyperstimulation animal model [170].

The metalloprotease neprilysin (NEP) also plays a role in apelin degradation. The “RPRL” motif, a sequence of amino acids surrounding the site of NEP proteolysis, is crucial for Apelin receptor binding and consequent physiological activity [27]. Creating analogues that are stable to NEP could lead to new, potent therapeutic peptides and provide a better understanding of the role of NEP in apelin breakdown. More stable apelin analogues were synthesized to counteract the NEP activity. Interestingly, the addition of “A2” modification to C-terminal also decreased the cleavage impact of ACE2 [87,177]. Results showed that the replacement of the amino acids arginine (Arg) and leucine (Leu) with azapeptides in apelin improved proteolytic stability towards NEP compared to native apelin isoforms [177]. In a later study, Fernandez et al. 2021 investigated the substitution of apelin amino acids with l-homoarginine (l-hArg) and l-cyclohexylalanine (l-Cha) and compared them to the most potent NEP-stabilized analogues produced by McKinnie group [178]. The results showed that apelin-17 analogues having l-Cha and l-hArg substitutions displayed enhanced stability compared to apelin-17 analogues with Arg or Leu substitutions. The former also demonstrated a prolonged lowering blood pressure effect in mice, while the effect on cardiac performance was not investigated [178].

NEP cleavage kinetics resembles human plasma kallikrein (KLKB1), a protease which cleaves apelin-17’s first three N-terminal amino acids only [30]. To overcome KLKB1 cleavage, Fischer et al. modified the N-terminal apelin-17 by either palmitoylation or PEGylation, thus significantly increasing plasma peptide half-life and decreasing blood pressure in mice [30]. More recently, the same authors synthesized other analogues of apelin-17 by incorporating N-methyl-arginine or α-methyl-arginine at position 14. Both analogues showed increased plasma stability compared to the native apelin-17, with the maintenance of improved blood pressure effect [179]. Moreover, the results showed that increasing the length of the PEG chain generally increases apelin plasma stability, and the addition of an N-terminal aromatic headgroup further boosts proteolytic resistance. Notably, the carbamate analogues had superior metabolic stability compared to the amides [179].

A combination of the modifications that affect each protease involved in apelin degradation might be investigated.

#### 4.1.2. Apelin Cyclization

Cyclization is another method to apply conformational restrictions to peptides in order to increase bioactivity, selectivity, and bioavailability, and the cyclized peptides are designed to occupy a comparable receptor ligand binding site. MM07 is a cyclic apelin mimetic peptide designed to extend circulating apelin half-life. Its cyclised N-terminal structure was designed to protect against enzymatic degradation by non-specific aminopeptidases and it was tested in first-in-human studies [24]. Results showed that MM07 was a more effective vasodilator and increased cardiac output than [Pyr1]-apelin-13 in both rodents and humans. Systemic effects of MM07 in rat showed that it caused a significant increase in cardiac output due to an inotropic effect comparable to commonly used inotropic drugs, without any evidence of chronotropic enhancement or hemodynamic instability [24]. Moreover, administration of MM07 in rats effectively reduced monocrotaline-induced increase in right ventricular pressure and hypertrophy, as well as monocrotaline-mediated changes in cardiac structure and function [181]. Although more in vivo investigations are required to deeply study the effect of this mimetic peptide, these recent results pave the way for heart failure potential therapeutic tool.

Another well-known technique for both increasing peptides’ proteolytic stability and controlling their biological activity is macrocyclization (formation of rings with more than 12 atoms), which has been effectively used against different targets, including proteases, protein–protein interactions, and GPCRs [184]. A series of macrocyclic analogues of apelin-13, built from [Pyr1]-apelin-13 using Fmoc strategy and ring-closing metathesis, were studied in HEK 293 cells using BRET-based biosensors. Results showed that this analogue had the strongest signalling for Gαi1, β-arrestin1, and β-arrestin2. Both the analogues obtained from modification of C-terminal exocyclic residue of apelin-13 and the analogues generated through a modification of endocyclic positions or Lys replacement of Arg residues showed a strong bias towards G-protein activation over β-arrestin signalling and had a binding affinity close to that of [Pyr1]-apelin-13. Another apelin analogue bearing a more flexible β-alanine spacer exhibited the same APJ affinity range of apelin-13 and compound carrying O-benzyl-Tyrosine substitution of Phe13. The investigation in hypertensive rats highlighted an effective hypotensive effect of this analogue similar to apelin-13 [182].

Other authors built new analogues with enlarged macrocycle size to improve APJ binding affinity, and they also showed similar properties to apelin-13 in terms of signalling and β-arrestin2 recruitment. The N-terminal portion was found to be important for ligand binding, while the ring size or linker type influenced the interaction between peptides and proteases. When tested in rats, the compounds, in which allylglycine was replaced by Pro, or Arg was substituted with Lys at N-terminus, showed similar hypotensive effects to apelin-13. The results of this study confirmed that macrocycle size and N-terminal portion play important roles in ligand binding, and the authors suggested that it can modulate both blood pressure and cardiac performance [168].

In a more recent study, two new macrocyclic analogues were created modifying linker and size of macrocycle: Nγ-allyl-Nγ-nosyl-α,γ-diamino-butanoic acid and Nπ-allyl-histidine linker placed at the apelin-13′s His8 position, which showed improved binding affinity and potency, respectively. Truncation of the peptide at C-terminal reduced the binding affinity, while changing the Nle11 position restored similar binding affinity to apelin-13. In addition, in an in vivo model an improvement in cardiac functions (i.e., fractional shortening and cardiac output) and a longer in vivo half-life were observed in rat plasma with respect to apelin-13 [183].

#### 4.1.3. Residue Modification of Apelin and ELA

Two apelin-17 analogues, P92 (generated by classic chemical substitutions in apelin-17) and LIT01-196 (produced by fluorocarbon chain addition to the N-terminal of apelin-17) displayed extended plasma half-life compared to parental apelin and sub-nanomolar affinity for APJ and showed full agonist activity for cAMP, ERK1/2 phosphorylation, β-arrestin recruitment, and APJ internalization [175]. Both analogues exhibited potential for the treatment of cardiovascular diseases by inhibiting the release of vasopressin into the bloodstream, increasing diuresis and decreasing arterial blood pressure [175]. Recently, the intravenous or subcutaneous administration of LIT01-196 reduced blood pressure in normotensive and hypertensive rats. Interestingly, LIT01-196 was more active than apelin-17 analogues in reducing blood pressure in hypertensive rats and was proposed as a potent long-lasting anti-hypertensive agent [176].

Another group synthesized the apelin-12-derived peptide MA12, having the addition of [N^α^MeArg1] moiety in the N-terminus and the replacement of easily oxidized Met10 by Nle, characterised by reduced proteolytic enzyme degradation. In rats, the addition of either MA12 or the natural NO vehicle significantly improved functional and metabolic recovery of the reperfused hearts and reduced membrane damage during reperfusion compared to the control group. However, the recovery of cardiac pump function was higher in the MA12 and natural NO vehicle groups compared to the control. Surprisingly, cardioplegia with MA12 showed a greater improvement of cardiac function compared to the natural NO vehicle [173]. More recently, the modified analogue [MeArg1 Nle10]-apelin-12 showed a three times significant increase in half-life compared to apelin-12, due to its methylation. The infusion of [MeArg1, Nle10]-apelin-12 for 10 min in 8-week Dox-treated rabbits had positive inotropic effects and reduced cardiomyopathy, without affecting heart rate. The cessation of the analogue peptide treatment, however, led to a reduction in heart function indices, leading to the necessity for further investigations at longer infusion time. Interestingly, the analogue peptide resulted as more effective than the apelin-12 in improving heart function [174].

Due to steric hindrance or conformational changes on [Pyr1]-apelin-13 brought on by the methyl group, the substitution of Gly, Met, Lys, His, and Ser had a significant decrease receptor activation. Only slight potency reductions were seen with all other N-Me analogues [171]. Recently, 40 variations of [Pyr1]-apelin-13 were synthesized, including backbone-alkylated dipeptides and modified amino acids [172]. The addition of various alkyl groups onto the N^α^ of Phe13 produced potent APJ ligands with affinities in the low nM range. The introduction of more extended and bulkier substituents resulted in compounds with similar binding affinities but reduced signalling potency for the β-arrestin 2 pathway. Incorporation of a positive charge on the C-terminal residue provided near-native binding associations and unique signalling profiles with partial agonism for β-arrestin 2 recruitment. One promising analogue, characterized by 1-Nal12 replacement with amino-indoloazepinone-Orn, had a high affinity for APJ, high Gαi1 activation, improved stability, and greatly decreased hypotensive effects [172].

The comparison of ELA with apelin-13 revealed that, for ELA, the C-terminal segment is also sufficient for binding APJ. In fact, alignment of apelin-13 and ELA sequences indicated that ELA and apelin-13 have comparable motifs, such as Pro30-Phe31 in ELA versus Pro12-Phe13 in apelin-13 at the C-terminus, His26 in ELA vs Lys8 in apelin-13, and the N-terminal Arg20 in ELA vs Arg2 in apelin-13 [185]. Nevertheless, the substitution of Phe31 with bulkier amino acids (such as Tyr (OBn), Bpa, or 1Nal) that considerably enhanced apelin-13 affinity for APJ when replaced to Phe13 residue did not provide comparable advantages to ELA, while if this replacement occurred in Pro32, the APJ binding affinity would be enhanced [180]. Therefore, the authors suggested that ELA and apelin-13 do not display similar structure-related activity. In fact, Val29 and His26 are crucial for ELA-APJ interaction, and Leu25 is more prone to substitution being structurally more exposed to environment [180]. These results are in line with Murza and colleagues who showed with alanine scan analyses that the C-terminal portion and His26 residues of ELA are crucial for binding and signalling, while for apelin-13, the key pharmacophores are located in the N-terminal [38].

The modification of ELA’s C-terminal residues had a significant impact on signalling on the Gα12 pathway [180]. The same authors showed in an in vivo model that the short ELA analogue generated by isosteric replacement of Met23 with norleucine (Nle) produced similar cardiac effects to the endogenous ligand but did not reduce blood pressure [180].

The proteolytic degradation of ELA in rat plasma and two fragments of different molecular weights, ELA(19-32) and ELA(22-32), were identified. ELA(19-32) consists of the last 14 amino acids of ELA and binds APJ, activates the Gαi1 and β-arrestin-2 signalling pathways, and induces receptor internalization. ELA and ELA(19-32) caused apelin-13-like hypotensive effects and increased left ventricular contractility in isolated ex vivo and in vivo hearts. Since this fragment rapidly underwent proteolytic degradation, the authors prolonged its half-life by N-terminal polyarginine and the cysteine residues (Pyr-R-R-C) removal, thus suggesting that it as a promising therapeutic agonist [38].

### 4.2. Antibody-Based/Bound APJ Agonists

Antibodies are widely used as therapeutic agents due to their ability to recognize and bind to specific receptors with high specificity and affinity. Nowadays, monoclonal antibodies have been utilized successfully in clinical treatment, despite the possibility of causing adverse immune-related reactions (Table 3).

In a recent study, researchers compared the structure of JN241 APJ-antagonist to that of the potent peptide agonist AMG3054 and generated a panel of JN241 mutants to convert JN241 from antagonist to agonist. Based on this evidence, authors hypothesized that the introduction of amino acids containing aromatic rings could fix the missing hydrophobic interactions and convert JN241 from antagonist to agonist. The tyrosine insertion between E104 and S105 in CDR3 of JN241 converted JN241 to a full agonist (JN241-9) with an EC50 of 36 and 47 nM in the cAMP assay and β-arrestin recruitment assay, respectively. Direct mutation of E104 or S105 to tyrosine or phenylalanine had no effect on antagonist-to-agonist conversion. Molecular modelling and simulation of APJ with JN241 or JN241-9 showed how the inserted tyrosine introduced additional hydrophobic interactions with APJ sub pocket residues, leading to an outward movement of TM6 as an early receptor activation event [186].

Antibody modifications can be considered to enhance the binding affinity, specificity, and stability of the antibody and to optimize its pharmacokinetic and pharmacodynamic properties for therapeutic applications.

Previously, the genetic fusion of human Domain Antibodies (dAbs) with serum albumin (AlbudAbs) was reported to increase the in vivo half-life of recombinant proteins [189]. MM202 is an apelin-modified APJ agonist conserving sequence similarity and functionality of endogenous apelin. MM202 and MM202-AlbudAb conjugate had high binding affinity to the human APJ and displayed functional activity in cell-based signalling assays, with either similar or greater potency than [Pyr1]-apelin-13. Both AlbudAb and MM202-AlbudAb conjugate bound to immobilized human serum albumin with high nanomolar affinity. In an acute cardiovascular study, the MM202-AlbudAb conjugate produced robust cardiovascular responses in normotensive male Sprague Dawley rats, including a reduction in left ventricular systolic pressure, an increase in cardiac contractility, and a decrease in heart rate [187].

The IgG-Fc-ELA protein fusion could also represent a therapeutic option for heart failure. Cardioprotective effects of IgG-Fc-ELA-21 fusion proteins were compared to those of parental peptide. Researchers found that the Fc-ELA-21 was expressed intact in transduced HEK293 cells, without the known furin proteinase-dependent ELA cleavage between position 22 and 23, displaying a half-life of approximately 44 h in mice after subcutaneous injection. The fusion protein showed significant improvements in heart function in rats after 4 weeks of daily injections. Rat treatment with the Fc-ELA-21 fusion protein increased angiogenesis, improved CM proliferation, and reduced apoptosis and heart fibrosis in the infarct area. In comparison, ELA-21 had a half-life of only 13 min and had no significant cardioprotective effects [188].

Lack of human clinical data on agonist peptides emphasizes the need for further research on the safety and efficacy of this promising approach as a new pharmacological option for cardiovascular disease treatment.

### 4.3. Side Effect and Delivery Issues

While the chemotactic and angiogenic activities of the native apelin and ELA peptides may have important implications in post-MI-related heart failure, they might, however, be detrimental in other conditions such as tumour growth [190]. In fact, it is noteworthy that the signalling pathways triggered in cardioprotection (i.e., MAPK and PI3K/AKT/mTOR) are also involved in cancer growth [191]. Moreover, more research is also needed in order to assess the potential renal and hormonal effects of APJ exogenous agonists, particularly if administered systemically. In fact, apelin reduces water reabsorption by acting at a central and renal level. In this context, plasma apelin and antidiuretic hormone (ADH) are inversely regulated for the maintenance of body fluid homeostasis. In the brain, apelin inhibits the release of ADH into the bloodstream from the posterior pituitary, while in the kidney, apelin regulates renal microcirculation and counteracts ADH-mediated water reabsorption in the collecting duct [192].

The systemic administration of both native and analogue APJ ligands may thus be potentially dangerous. Additionally, the successful outcome of conventional systemic APJ ligand administration procedures is hampered by various issues including the absence of targeting capability to affected sites, poor control of sustained delivery along the necessary therapeutic time, the need for a high peptide concentration at the target site, and possible peptide instability. These peptides could be rather delivered using engineered nanotechnologies as drug delivery systems [193,194]. In this context, Serpooshan and colleagues already performed in 2015 a [Pyr1]-apelin-13 nanodelivery by using a liposomal nanocarrier system, and the intraperitoneal injection of [Pyr1]-apelin-13 nanocarriers prevented cardiac disfunction in a TAC mouse model. In comparison to the commercially available [Pyr1]-apelin-13, such nanocarriers incorporating PEG polymer exerted significantly increased apelin delivery and sustained release both in vitro and in vivo [195]. Similarly, nanotechnologies may be advantageous also for analogue molecule delivery.

Since primary cardiac tumours are exceptionally rare, nanotechnologies and /or biomaterials can also be exploited for the in situ release of APJ analogues, allowing the use of lower dosages compared to systemic administration, causing minor plasma fluctuations, and reducing adverse effects [196]. In a recent study, a microporous annealed particle drug-delivery hydrogel, able to assemble once injected into the heart of infarcted rats forming a porous scaffold, showed promising positive effects in the field of heart regeneration. However, this hydrogel requires a longer-term study as it showed only partial degradation in vivo after 5 weeks [197]. These notes provide the rationale to promote the development of drug-delivering materials able to degrade over time, such as bioresorbable scaffolds, in order to reduce side effects [198]. Despite nanotechnologies and biomaterials are promising therapeutic tools, they still have some limitations due to high costs and safety problems [199].

The use of local administration and biomaterial-mediated controlled analogue release may represent a strategy to overcome potential systemic side effects, allowing in situ molecule delivery and reduction of treatment dosage. Additionally, the use of APJ ligand-derived analogues may have the advantage of low production costs and reduced immune response risks.

## 5. Conclusions and Future Perspectives

A large number of evidence supports the idea that the apelin/ELA-APJ axis can protect the cardiovascular system against the occurrence of hypertension or MI and their progression to heart failure. The modulation of the APJ-related pathways could potentially provide an excellent therapeutic target for cardiovascular diseases. The multiple beneficial effects and the complexity of the APJ-related signalling pathways make convenient an approach based on full receptor activation, rather than on activation of a single APJ-related cellular pathway of interest. Since endogenous APJ ligands are characterized by a short half-life, various research groups are trying to modify the native molecule to obtain analogues as potential therapeutic tools for clinical application. Most of the studies have focused on improving the plasma stability and the APJ binding affinity through a variety of methods, including PEGylation, acylation, palmitoylation, cyclization, as well as addition or substitution with unnatural amino acids. Nevertheless, only a few in vivo studies evaluated improvement in cardiac function and/or a reduction in arterial pressure after analogue administration in animal models. Moreover, whether these new peptides are capable of mimicking the chemotactic and angiogenic activities of native ones has still to be tested in vivo. Additional studies are needed to assess the long-term safety and efficacy of these analogues in humans, as well as to identify any potential side effects or drug interactions before translating this new strategy into clinical practice. Due to the highly variable degree of translational development, it is not easy at the present time to select the most promising peptide candidates. Although some groups have already tested their analogues in pathologic preclinical models, the heterogeneity of employed models, the different follow-up timing, and the variety of analysed endpoints still preclude a direct comparison leading to the most promising candidate(s) selection. In the future, employment of nanotechnologies and biomaterials will result as very useful for in situ administration of these analogues to the post-MI heart with the important advantages of increasing in loco bioavailability and limiting systemic side effects.

## Figures and Tables

**Table 1:** Main chain modifications of APJ ligands, based on [Pyr1]-apelin-13, apelin-12, apelin-17, or ELA peptide, and their effects on stability, binding affinity, and cardiovascular properties.

APJ Ligand	Substituted Residues	Introduced Residues	Effects	Ref
[Pyr1]-apelin-13	Leu5, Ser 6	-	↑ plasma protein stability	[167]
	Phe13	[(L-α-Me) Phe]	↓ Pro12-Phe13 hydrolisis	
	Pro12-Phe13	conformational constrained amino acids	↑ binding affinity ↑ plasma protease stability	[168]
		Aia -Phe 1Nal-α,α-dibenzylglycine	↑ Gα12 pathways ↑ affinity and resistance to ACE2 cleavage ↑ in vitro and in vivo pharmacokinetics	[169]
	Phe13	phenyl groups at the ortho-, meta-, and para-positions of Phe13	slight effect on APJ binding affinity	
		(L-α-Me) Phe or p-benzoyl-L-Phe (Bpa)	↑ APJ functional selectivity for Gαi proteins no effect on cardiac contractility in healthy rats ↑ binding affinity vs. [Pyr1]-apelin-13 ↑ resistance to ACE2 cleavage ↓ hypertrophy and fibrosis	[170]
	N-Teminus (pGlu)	palmitic acid, (at Pro12) Aib, Nle	↑ half-life (29 h in rat plasma)	[171]
	N-Terminus	Alkylated dipeptide	↑ affinity in the low nM range	[172]
		extended and bulkier substituents	binding affinity close to [Pyr1]-apelin-13 ↓ signalling potency for the β-arrestin 2 pathway	
	C-Terminus	positive charge	binding affinity close to [Pyr1]-apelin-13 partial agonism for β-arrestin2 recruitment	
	1NaI12	amino-indoloazepinone-Orn	↑affinity for APJ ↑ Gαi1 activation, ↑ stability ↓ hypotensive effects	
apelin-12	N-terminus Met10	(N^α^Me) Arg Nle10	↑ proteolytic stability ↑ cardiac function ↓ membrane damage	[173,174]
apelin-17	Classical substituted	P92	↑ plasma half-life sub-nanomolar affinity for APJ APJ internalization ↑ diuresis ↓ arterial blood pressure	[175]
	LIT01-196	fluoroaddiction N-terminal		
			↓ blood pressure in normotensive and hypertensive rats	[176]
	Arg, Leu	azapeptides	↑ proteolytic stability vs. NEP	[177]
	l-hArg, l-Cha	-	↑ proteolytic stability vs. NEP prolonged lowering blood pressure effect in mice	[178]
	N-terminus	palmitoylation PEGylation	↑ plasma peptide half-life ↓ blood pressure in mice	[30]
		aromatic head groups carbonate analogues	↑↑ proteolytic stability	[179]
ELA	Pro32	Tyr (OBn), Bpa, or 1Nal	↑ binding affinity vs. ELA	[180]
	N-terminus	pyroglutamic acid (Pyr) residue to ELA(19-32)	↓ arterial pressure ↓ inotropic effects on the heart ↑ half-life	[38]

**Table 2:** Peptides obtained by apelin cyclization and their effects on plasma stability, binding affinity, and cardiovascular properties.

Cyclized Peptide	Modified Residues	Cycle Position	Effects	Ref
MM07	N-terminus	N-terminal cycle (MM07)	↑ vasodilatation ↑ cardiac function vs. [Pyr1]-apelin-13	[24]
			↑ cardiac function ↓ hypertrophy	[181]
Macrocycle analogues	G-P-M−P-F	[X-P-Nle-P-X] [B1-P-Nle-P-X] [B1-P-Nle-P-Xd]	↑ plasma peptide half-life ↑ binding affinity vs. apelin-13 powerful modulators of cardiovascular system	[168]
	C-terminal	C-thermal exocyclic residues endocyclic residues	↑ G-protein activation on over-arresting signalling binding affinity close to [Pyr1]-apelin-13	[182]
	Internal position			
		β alanine spacer	Same APJ affinity of apelin-13 ↓ hypotensive effect similar to apelin-13	
	apelin-13′s His8 position	Nγ-allyl-Nγ-nosyl-α,γ-diamino-butanoic acid Nπ-allyl-histidine linker	↑ binding affinity ↑ potency	[183]

**Table 3:** Antibody based/bound APJ agonists and their effects on plasma stability, binding affinity, and cardiovascular properties.

Agonist	Substituted Residues	Introduced Residues	Effects	Ref
JN241-9	between E104 and S105 to JN241	tyrosine	agonist activity for cAMP β-arrestin recruitment	[186]
MM202 (apelin-modified)	Met	Nle and Fluroaddition-C-terminal	high binding affinity vs [Pyr1]-apelin-13 = or ↑ potency than [Pyr1]-apelin-13	[187]
MM202-AlbudAb			= or ↑ potency than [Pyr1]-apelin-13	
		MM202 and AlbudAb fusion	↑ cardiac function ↓ heart rate	
Fc-ELA-21	-	Fc and ELA fusion	↑ angiogenesis ↓ apoptosis ↑ cardiomyocyte proliferation ↓ heart fibrosis	[188]

## Data Availability

Not applicable.
